# Transurethral resection of the prostate provides more favorable clinical outcomes compared with conservative medical treatment in patients with urinary retention caused by benign prostatic obstruction

**DOI:** 10.1186/s12877-018-0709-3

**Published:** 2018-01-16

**Authors:** Yu-Hsiang Lin, Chen-Pang Hou, Tien-Hsing Chen, Horng-Heng Juang, Phei-Lang Chang, Pei-Shan Yang, Chien-lun Chen, Ke-Hung Tsui

**Affiliations:** 1 Department of Urology, Chang Gung Memorial Hospital-Linkou, 5 Fu-Shing Street, Kweishan, Taoyuan, 333 Taiwan, Republic of China; 2 School of Medicine, Chang Gung University, 259 Wen-Hwa 1 st Road, Kweishan, Taoyuan, Taiwan, Republic of China; 3 Department of Anatomy, School of Medicine, Chang Gung University, Kwei-shan, Tao-Yuan, Taiwan, Republic of China; 4 Graduate Institute of Clinical Medical Sciences, College of Medicine, Chang Gung University, Taoyuan, Taiwan, Republic of China; 5 Division of Cardiology, Department of Internal Medicine, Chang Gung Memorial Hospital, 222, Maijin Road, Keelung, Taiwan, Republic of China

**Keywords:** Alfa-blocker, Benign prostate hyperplasia, Urine retention, Outcome, Prostatectomy

## Abstract

**Background:**

To evaluate the long-term surgical outcomes of patients with urinary retention (UR) caused by a benign prostatic obstruction (BPO) who underwent transurethral resection of the prostate (TURP), and compare their outcomes with those of patients who received medication without surgical intervention.

**Methods:**

This retrospective cohort study analyzed claims data collected during the period of 1997–2012 from Taiwan’s National Health Insurance Research Database. We examined geriatric adverse events among patients who had received a diagnosis of symptomatic benign prostatic hyperplasia and whom experienced UR, and compared those who received TURP and medication only. Primary outcomes included urinary tract infection (UTI), UR, inguinal hernia, hemorrhoids, stroke, acute myocardial infarction, and bony fracture. We excluded patients who had concomitant prostate cancer, bladder cancer, or a long-term urinary catheter indwelling, as well as those who did not receive α-blocker medication regularly. Those aged <50 or >90 years were also excluded. The enrolled patients were categorized into TURP (*n* = 1218) and medication only (*n* = 795) groups. After 1:1 propensity score matching, we recorded and compared patients’ characteristics, postoperative clinical outcomes, and geriatric adverse events.

**Results:**

The TURP cohort had a lower incidence of UTI and UR during the postoperative follow-up period from 2 months to 3 years than did the medication only group (20.7% vs. 28.9% and 12.5% vs. 27.6%, respectively, *p* < 0.001). The life-long bone fracture incidence was also lower in the TURP cohort (7.9% vs. 9.2%, *p* = 0.048). The incidence of other outcomes during the postoperative follow-up period did not differ between the two groups.

**Conclusions:**

Compared with conservative treatment, TURP provides more favorable clinical outcomes in patients with UR caused by BPO. Patients who underwent TURP had a lower risk of UTI, repeat UR episodes, and emergent bony fracture. Thus, early surgical intervention should be considered for such patients.

## Background

Benign prostatic hyperplasia (BPH) affects approximately 210 million men globally and is a major cause of lower urinary tract symptoms (LUTSs) in aging men [1]. LUTSs negatively affect patients’ quality of life and cost the US healthcare system more than $4 billion each year [2]. One study estimated that 50% and 75% of men have histological evidence of BPH by the age of 50 and 80 years, respectively, with approximately 50% of them having clinically significant symptoms [3]. The sequelae of BPH include a decreased urinary flow and advancing voiding and storage symptoms; these may eventually result in acute or chronic urinary retention (UR) [4]. Although men with acute UR caused by BPH have an increased chance of returning to normal voiding if treatment with α-1 blockers is started at the time of catheter insertion [5], 24%–42% of patients elect to receive surgical intervention instead [6, 7]. According to the updated guidelines, surgical intervention is an appropriate treatment for patients with moderate-to-severe LUTSs and for patients who have developed acute UR or other BPH-related complications [8]. Surgical treatment is often effective and prevents the need for indwelling or intermittent catheterization in the future [9, 10]. However, in a previous study, 79% of patients received α1-blockers before catheter removal, and most of them could void successfully without requiring an indwelling catheter [11]. To the best of our knowledge, very few studies have compared long-term treatment outcomes between transurethral resection of the prostate (TURP) and medical treatment in patients with BPO who experience UR. Therefore, using data from the National Health Insurance Research Database (NHIRD) of Taiwan, we conducted a nationwide observational cohort study to investigate the surgical outcomes of such patients and compare the long-term treatment outcomes between patients who received medication only and those who underwent surgical intervention.

## Methods

### Data source

We used data from the Longitudinal Health Insurance Database 2000 (LHID2000) in this study. This database contains the claims data of beneficiaries enrolled in the National Health Insurance (NHI) program of Taiwan; to date, more than 4000 research articles have been published using the NHIRD [12]. The LHID2000 includes the claims data of 1000,000 individuals randomly sampled from the entire population enrolled in the NHI program (a total of 23.75 million people) in 2000. The demographic characteristics (i.e., age and sex) between the populations derived from the NHIRD and LHID2000 are not significantly different.

### Study design

**Fig. 1:**
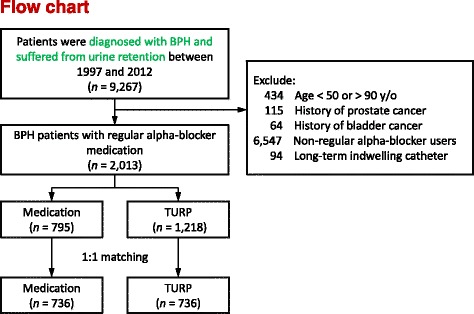
Flowchart for patient inclusion in this study

We identified patients who had received a diagnosis of BPH (International Classification of Diseases, Ninth Revision, Clinical Modification [ICD-9-CM] code 600.xx) and had visited the emergency department or an outpatient clinic presenting with UR between January 1, 1997, and December 31, 2012. All identified patients had received α-blockers for at least 6 months before the UR episode. UR is defined as indwelling Foley catheterization (Taiwan NHI code 47014C) or intermittent catheterization (Taiwan NHI code 47013C). If patients underwent TURP after BPH with an UR episode, the index date was defined as the discharge date after TURP; otherwise, the index date was defined as the date of the UR episode. Patients who met the following criteria were excluded: (1) age < 50 years or >90 years, (2) a diagnosis of prostate cancer (ICD-9-CM code 185.xx) or bladder cancer (ICD-9-CM code 188.xx) before the index date, (3) regularly receiving α-blockers for more than 6 months before the index date, and (4) long-term use of an indwelling urinary catheter. Finally, 2013 patients were included, of which 1218 underwent TURP for UR and 795 received medication without surgical intervention. After 1:1 propensity score matching, we subgrouped the patients into two cohorts: the TURP cohort (*n* = 736) and the medication only cohort (*n* = 736). Finally, patients’ characteristics, postoperative clinical outcomes, and geriatric adverse events were recorded and compared. The flow chart for patient enrolment is illustrated in Fig. 1.

### Comorbidity detection

The following comorbidities were included in this study: diabetes mellitus (ICD-9-CM code 250.xx), hypertension (ICD-9-CM codes 401.xx–405.xx), dyslipidemia (ICD-9-CM code 272.4), chronic obstructive pulmonary disease (ICD-9-CM codes 491.xx, 492.xx, and 496.xx), Parkinson disease (ICD-9-CM code 332.xx), chronic renal disease or renal failure (ICD-9-CM codes 584.xx and 585.xx), ischemic heart disease (ICD-9-CM codes 410.xx–414.xx), stroke (ICD-9-CM codes 430.xx–437.xx), and heart failure (ICD-9-CM 428.xx). The presence of a comorbidity was ascertained when there was at least one claim of inpatient admission or two claims of outpatient visits 1 year prior to the index date.

### Outcome detection

The outcomes compared in this study were urinary tract infection (UTI) and UR. UTI was defined as hospitalization or an emergency department or outpatient visit with a UTI-related diagnosis (ICD-9-CM codes 599.0× and 595.0×) and antibiotic prescription. UR was defined as the occurrence of either an intermittent catheterization or indwelling Foley catheterization. Other geriatric adverse events that were compared included inguinal hernia (Taiwan NHI codes 75606B, 75607C, 75613C, 75614C, and 75610B), hemorrhoids (Taiwan NHI codes 74406C, 74407C, 74410C, 74411C, 74412C, and 74417C), stroke (ICD-9-CM codes 430.xx–437.xx), and acute myocardial infarction (ICD-9-CM code 410.xx). We also compared the incidence of emergent bone fracture, which was defined as visiting the emergency department or hospitalization with a principal diagnosis of skeletal fracture (ICD-9-CM code 805.xx-829.xx), between the two cohorts, as well as the incidence of urological malignancies after the index date, including prostate cancer (ICD-9-CM code 185.xx) and bladder cancer (ICD-9-CM code 188.9×). The malignancy-related diagnosis was verified as the possession of a catastrophic illness certificate (CIC) card.

### Statistical analyses

To reduce the possible selection bias and rule out confounding factors, we matched each patient in the TURP group with a counterpart in the medication only group through propensity scoring [13]. The distribution of demographic and clinical characteristics in the TURP and medication only groups was compared using a chi-square test (for the categorical variables) and an independent sample t test (for the continuous variables). The risk of geriatric or urological malignancy-related adverse events during follow-up was also compared between the study groups by using a Cox proportional hazard model. Additionally, the cumulative survival of bone fracture in the two groups was estimated using the Kaplan–Meier method. Propensity score matching and all statistical analyses were performed using SAS software, version 9.4 (SAS Institute, Cary, NC, USA).

## Results

### Study population

After 1:1 propensity score matching, we grouped the patients into two cohorts: the TURP cohort (*n* = 736) and medication only cohort (*n* = 736) (Table 1). The mean age and follow-up duration of the patients in the TURP and medication only cohorts were 74.2 years (standard deviation [SD] = 7.9 years) and 4.2 years (SD = 3.4 years), respectively. The mean age and prevalence of comorbidities did not differ significantly between the two cohorts, and the Charlson comorbidity index [14] of the two cohorts was comparable.

**Table 1:** Patient characteristics after propensity score matching

Variable	TURP group (*n* = 736) *n* (%)	Medication group (*n* = 736) *n* (%)	*P* value
Age (years)	74.1 ± 7.5	74.3 ± 8.4	0.528
Comorbidity			
Diabetes mellitus	234 (31.8)	221 (30.0)	0.463
Hypertension	467 (63.5)	455 (61.8)	0.518
Hyperlipidemia	111 (15.1)	105 (14.3)	0.659
Chronic obstructive pulmonary disease	155 (21.1)	166 (22.6)	0.487
Parkinsonism	34 (4.6)	40 (5.4)	0.474
Chronic kidney disease	114 (15.5)	116 (15.8)	0.886
Ischemic heart disease	196 (26.6)	179 (24.3)	0.309
Stroke	132 (17.9)	137 (18.6)	0.736
Heart failure	60 (8.2)	63 (8.6)	0.778
Charlson Comorbidity Index	1.3 ± 1.2	1.2 ± 0.9	0.425

*Abbreviations*: *TURP* transurethral resection of the prostate

### Postoperative adverse events

Geriatric adverse events were compared between the two cohorts to evaluate their association with postoperative adverse events during the 3-year follow-up period (Table 2). Overall, the TURP group had a lower risk of UTI (hazard ratio [HR], 0.62; 95% confidence interval [CI], 0.50–0.76) and UR requiring catheterization (HR, 0.35; 95% CI, 0.27–0.45) during the 2 months to 3 years postoperative period. The TURP group also had a lower risk of bone fracture during the postoperative 3-year follow-up period (HR, 0.58; 95% CI, 0.37–0.90).

**Table 2:** Geriatric adverse events during the 3-year follow-up period

Variable	TURP (*n* = 736) n (%)	Medication (*n* = 736) n (%)	TURP vs. Medication	
			HR (95% CI)	*P* value
UTI (post-op 2 months – 3 years)	152 (20.7)	213 (28.9)	0.62 (0.50, 0.76)	<0.001
UR (post-op 2 months – 3 years)	92 (12.5)	203 (27.6)	0.35 (0.27, 0.45)	<0.001
Inguinal hernia	18 (2.4)	17 (2.3)	0.96 (0.50, 1.87)	0.914
Hemorrhoids	9 (1.2)	9 (1.2)	0.92 (0.37, 2.33)	0.867
Stroke	28 (3.8)	25 (3.4)	1.01 (0.59, 1.73)	0.985
AMI	10 (1.4)	11 (1.5)	0.77 (0.33, 1.82)	0.550
Fracture	31 (4.2)	48 (6.5)	0.58 (0.37, 0.90)	0.016

*Abbreviations*: *TURP* transurethral resection of the prostate, *HR* hazard ratio, *CI* confidence interval, *AMI* acute myocardial infarction

**Fig. 2:**
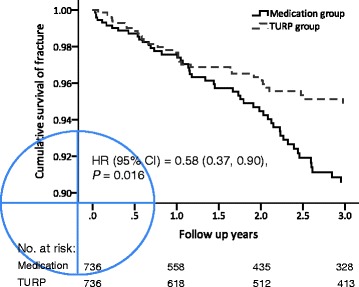
Kaplan–Meier plots showing the cumulative survival of emergent skeletal fracture in patients

Table 3 presents a comparison of life-long geriatric adverse events between the two groups. Although the life-long incidence of inguinal hernia and hemorrhoids was comparable, the TURP group had a lower risk of life-long bone fracture (HR, 0.70; 95% CI, 0.49–0.996). Fig. 2 shows the cumulative survival of bone fracture in the two groups, which indicates that the TURP group had a lower risk of bone fracture than did the medication only group.

**Table 3:** Comparison of life-long geriatric adverse events

Variable	TURP (*n* = 736) n (%)	Medication (*n* = 736) n (%)	TURP vs. Medication	
			HR (95% CI)	*P* value
Inguinal hernia	27 (3.7)	32 (4.3)	0.70 (0.42, 1.17)	0.169
Hemorrhoids	13 (1.8)	15 (2.0)	0.74 (0.35, 1.55)	0.425
Stroke	51 (6.9)	42 (5.7)	1.03 (0.68, 1.54)	0.906
AMI	21 (2.9)	15 (2.0)	1.13 (0.58, 2.20)	0.719
Fracture	58 (7.9)	68 (9.2)	0.70 (0.49, 0.996)	0.048

*Abbreviations*: *TURP* transurethral resection of the prostate, *HR* hazard ratio, *CI* confidence interval, *AMI* acute myocardial infarction

### Urological malignancy-related adverse events

Urological malignancy-related adverse events were compared between the two groups at the end of the follow-up period (Table 4). After the index date, prostate cancer was detected in 18 patients (2.4%) in the TURP cohort and in 19 patients (2.6%) in the medication only cohort during the follow-up period (HR, 0.79; 95% CI, 0.41–1.50). Furthermore, bladder urothelial carcinoma was detected in 5 patients (0.7%) in the TURP cohort and in 10 patients (1.4%) in the medication only cohort during the follow-up period (HR, 0.41; 95% CI, 0.14–1.19). These findings suggest that TURP cannot reduce the incidence of prostate and bladder cancer in patients with BPO who experienced an UR episode.

**Table 4:** Urological malignancy-related adverse events at the end of follow-up

Variable	TURP (*n* = 736)	Medication (*n* = 736)	TURP vs. Medication	
			HR (95% CI)	*P* value
Prostate adenocarcinoma	18 (2.4)	19 (2.6)	0.79 (0.41, 1.50)	0.463
Bladder urothelial carcinoma	5 (0.7)	10 (1.4)	0.41 (0.14, 1.19)	0.102

*Abbreviations*: *TURP* transurethral resection of the prostate, *HR* hazard ratio, *CI* confidence interval

## Discussion

All clinicians should particularly focus on BPH and BPO, because 50% of men develop pathological BPH at the age of 51–60 years [15]. In the United States, the estimated risk of a 50-year-old man with BPH undergoing therapeutic intervention (surgical or medical treatment) in his lifetime is approximately 40% [16]. A 3-year, multicenter, randomized controlled trial investigated patients with moderate BPH symptoms who were treated through either watchful waiting or TURP. In this trial, 24% and 2.9% of men in the watchful waiting arm crossed over to receive surgical intervention developed UR, respectively [17]. UR, one of the common complications of BPH and BPO, is a distressing urological emergency that seriously affects patients’ health and quality of life. Among men aged 70–79 years with BPH and moderate-to-severe LUTSs (IPSS score > 7), the incidence of UR was determined to be approximately 34.7 per 1000 person-years [18]. Another large-scale study that evaluated an ethnically diverse group of males in the United States reported that the observed incidence of BPH-associated UR increased substantially between 2007 and 2010 [19].

In men with BPH, risk factors for UR include advanced age, severe LUTSs, increased prostate volume, decreased urinary flow rate, and prostate-specific antigen level > 2.5 [20]. Three factors predominate the pathophysiological mechanisms of UR: outflow obstruction, neurological impairment, and an inefficient detrusor muscle [21], among which outflow obstruction is the most common cause [22]. Another urodynamic study on UR reported that outflow obstruction may develop secondary to the interruption of sensory or motor nerve supply to the detrusor muscle, incomplete relaxation of the urinary sphincter mechanism, or inefficient contraction of the bladder detrusor muscle [23].

Once acute UR occurs, the initial management includes immediate decompression of the urinary bladder through urethral Foley catheterization or indwelling suprapubic cystostomy if urethral catheterization is not possible [24–26]. Although UR is one of the absolute indicators for surgical treatment in patients with BPH/BPO [27], TURP is not the first choice of treatment in daily practice because of its potential risks and complications. Instead, α-blockers, which improve BPO in men with LUTSs, are regarded as the first-line treatment for BPO [28]. α-Blockers can result in a successful trial without catheter (TWOC) in patients with acute UR. Some urologists offer a trial of voiding to patients with acute UR, and one study reported that patients voided successfully by 12 weeks after TWOC without surgical treatment [29]. Another study reported that 48% of patients with acute UR had a successful TWOC when they were administered an α-blocker (Tamsulosin), whereas only 26% of patients had a successful trial when no drug was administered [30]. Elsewhere, researchers indicated that after initial catheterization, 72.8% of men had a successful TWOC after a median of 3 days of catheterization, of which 79% had received an α1-blocker (Alfuzosin) before catheter removal [11].

Although treatment with α-blockers without surgical intervention can result in a successful TWOC in patients with acute UR, studies have yet to demonstrate the long-term clinical outcomes of these patients. Therefore, the present study compared long-term clinical outcomes between patients who had received TURP and those who had received medication only by using data from Taiwan’s NHIRD. Before comparing the clinical outcomes of the two cohorts, we performed 1:1 propensity score matching [13] to ensure that the characteristics of the two groups were similar and more objective data could be obtained. Therefore, the distribution of age, incidence of preoperative comorbidities, and Charlson comorbidity index did not differ significantly between the two groups (Table 1).

Moderate-to-severe LUTSs considerably affect all parameters of quality of life for aging men [31], and appropriate management is warranted. TURP is a safe and effective surgical procedure for men with BPH and moderate-to severe LUTSs. TURP can even achieve favorable outcomes in stroke and DM patients with symptomatic benign prostate hyperplasia [32, 33]. All of the patients included in our study had received α-blockers for at least 6 months before experiencing an UR episode. Our results showed that clinical outcomes were more favorable in the TURP group compared with the medication only group, because the TURP group had a lower risk of UTI and UR. Furthermore, the TURP group had a lower incidence of future emergent skeletal fracture during both postoperative 3-year follow-up and life-long observation periods.

Nocturia is not only the leading cause of sleep fragmentation in older adults [34] but also a crucial risk factor for falls among men older than 65 years [35]. A study based on the Japanese National Health Insurance system reported that elderly individuals with nocturia had a higher risk of fracture and death than did those without nocturia [36]. Another study also reported an association of nocturia with a higher risk of comorbidities, such as bone fracture, diabetes, and coronary disease, and thus a higher risk of mortality among elderly individuals [37]. In patients who had experienced acute UR, TURP probably resulted in more favorable treatment outcomes compared with medication alone in terms of LUTSs, including nocturia. LUTSs were relieved once a patient received TURP. TURP appears to reduce the urge and prompt sensation to void and the number of times a patient gets up to visit the toilet at night, thus preventing them from the risk of falls. On the other hand, orthostatic hypotension is an independent risk factor for recurrent falls among the elderly [38]. After receiving TURP, the chance of a patient taking alpha-blockers would probably decrease, thereby reducing the possibility of fall caused by postural hypotension, which is the possible side effect of alpha-blockers.

In this study, we also investigated whether the medication only group had a higher future incidence of urological malignancy. Given that high post-voiding residual urine, repeat UTI, chronic bladder inflammation, and chronic UR all increase the urothelial exposure to carcinogens [39], we hypothesized that the medication only group would have a higher future incidence of bladder urothelial carcinoma. However, our data revealed that the incidence of both bladder urothelial carcinoma and prostate adenocarcinoma was identical in the two cohorts. This may be because the number of patients with malignancy in this study was too small to observe any statistical difference.

This study has some limitations that were inherited from the data structure of the NHIRD. First, this database does not provide detailed personal information, such as laboratory parameters, alcohol consumption, cigarette use, and exercise, which are confounding variables that influence LUTSs and bladder urothelial carcinoma. Some important reports like pre-operative prostate volumes and the urodynamic studies of the patients were not obtained in this study, either. Second, we used strict dichotomy to divide our study population into two groups: the TURP and medication only groups. Thus, we could not assess whether the time length from acute UR to surgery or whether the number of UR episodes affected treatment outcomes. Third, the use of prostatic vaporization (or ablation by laser), which is not reimbursed by the Taiwan NHI, has only become increasingly common in the last decade [40]. Thus, patients receiving prostate laser treatment were not included in this database. However, despite these limitations, this is the first study to compare the long-term treatment outcomes of TURP and medication only for patients who experience acute UR. Thus, we believe this is innovative and valid research.

## Conclusions

Although treatment with α-blockers without surgical intervention can result in a successful TWOC in patients with BPH/BPO who experience acute UR, TURP provides more favorable long-term clinical outcomes. The patients who received TURP had a lower risk of UTI, repeat UR episodes, and emergent bony fracture in the future than did those who received medication alone. We conclude that early surgical intervention should therefore be considered for such patients.

## Abbreviations

BPH: Benign prostate Hypertrophy
BPO: Benign prostatic obstruction
LUTS: Lower urinary tract symptoms
NHI: National Health Insurance
TURP: Transuretrhal resection of prostate
UR: Urine retention
